# Very-late-onset cytomegalovirus disease: a case-report and review of the literature

**DOI:** 10.1186/s13104-017-2532-x

**Published:** 2017-06-13

**Authors:** Hania Burgan, Gael Gosteli, Marc Giovannini, Reto Lienhard, Olivier Clerc

**Affiliations:** 1 Department of Internal Medicine and Infectious Diseases, Hôpital Neuchatelois-Pourtalès, Maladière 45, 2000 Neuchâtel, Switzerland; 2 Private Practice, La Chaux-de-Fonds, Switzerland; 3 ADMED Microbiology, La Chaux-de-Fonds, Switzerland

**Keywords:** CMV disease, Kidney transplant, Prophylaxis, Valganciclovir

## Abstract

**Background:**

Cytomegalovirus (CMV) infection remains one of the most common and feared complications of transplantation, justifying prophylaxis or preemptive strategies guided by donor and recipient CMV serostatus. In case of seronegative donor and recipient (D−/R−), no prophylaxis is recommended. Late-onset CMV disease is usually defined as occurring after prophylaxis discontinuation in D+/R− transplant patients.

**Case presentation:**

We are reporting the case of a D−/R− kidney Caucasian transplant recipient presenting with CMV primoinfection 12 years after renal transplant, and discuss the role of a secondary prophylaxis so late after transplantation.

**Conclusions:**

Primary infections leading to late-onset CMV disease in transplant patients remain rare. Recurrent disease has been described in as many as one-third of these patients. A systematic secondary prophylaxis in this particular group of patients is questionable.

## Background

Cytomegalovirus (CMV) infection remains one of the most common and feared complications of transplantation, justifying prophylaxis or preemptive strategies guided by donor and recipient CMV serostatus. In case of seronegative donor and recipient (D−/R−), no prophylaxis is recommended. Late-onset CMV disease is defined as occurring after prophylaxis discontinuation in D+/R− transplant patients [1]. In this article, we are reporting the case of a D−/R− kidney transplant recipient presenting with CMV primoinfection 12 years after renal transplant, and reviewed similar cases, regarding the role of antiviral treatment and secondary prophylaxis in such cases.

## Case presentation

A 69-year-old Caucasian male, who had received kidney transplantation 12 years earlier because of polycystic renal disease, was admitted to La Chaux-de-Fonds Hospital, Switzerland, with severe fatigue, diarrhea and loss of appetite, lasting for 2 weeks. Current immunosuppressive regimen consisted of cyclosporine, mycophenolate mofetil (500 mg 2/day) and low dose prednisone (5 mg daily). Severe chronic renal failure (glomerular filtration rate 15 ml/min) was attributed to immunosuppressive treatment and a previous episode of humoral graft rejection 5 years earlier. One week before hospital admission, the patient had had a left-ear basocellular carcinoma resection.

The patient presented a diminished general condition, mild hypotension, but no fever. On clinical examination, the abdomen was tender without signs of local peritoneal irritation. The rest of the physical examination was unremarkable. The day after admission, the patient developed fever with a peak temperature of 37.8 °C, and transient psychomotor slowing with persisting hiccup.

**Fig. 1:**
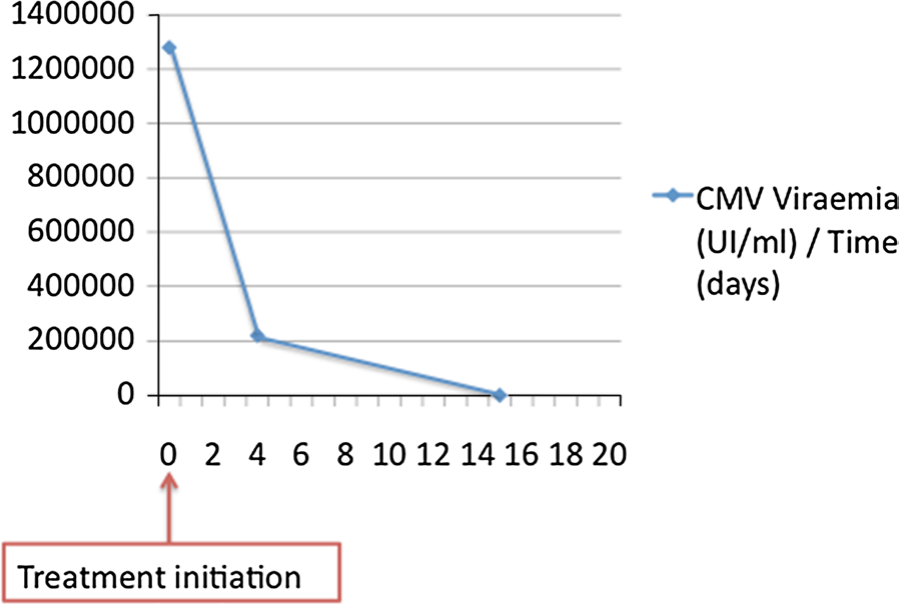
Evolution of CMV viral load under treatment

The initial blood cell count was within normal range (leucocytes 9.2 g/l), and blood chemistry revealed acute renal failure (creatinine 577 µmol/l) of pre-renal origin as well as slightly elevated liver enzymes with predominant cholestasis. Stool examination for *Salmonella*, *Shigella*, *Campylobacter* and *Clostridium difficile*, parasitologic examination and PCR for Norovirus infection came back negative. Initial CMV serology was positive for IgM only (1.9 U/ml, (0.7–0.9), Vidas, Biomerieux, France), whereas the previous serology 1 year earlier had been negative. Concomitant CMV viral load (Fig. 1) was elevated to 1′280′000 UI/ml (Cobas Taqman, Roche, Switzerland). Acute seroconversion was confirmed 10 days later with increasing IgM titer (2.4 U/ml) and newly detectable IgG titer (9 UA/ml, limit 4–6, Vidas, Biomerieux, France). Blood cultures returned transiently positive for *methicillin*- *susceptible*-*Staphylococcus aureus* (MSSA), as was a left-ear surgical site excision local swab.

**Fig. 2:**
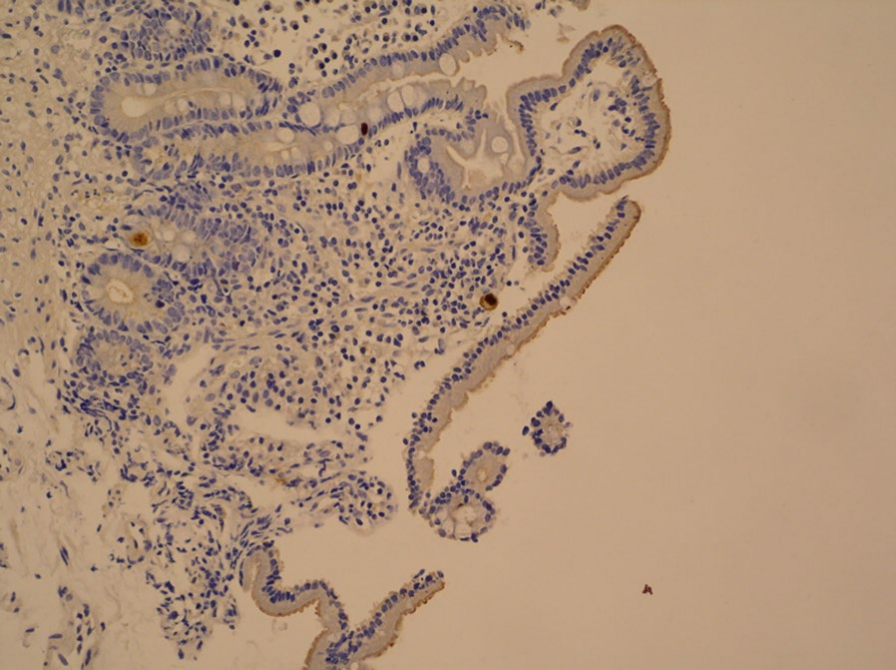
Rare CMV positive cells in duodenal mucosa (immunostaining)

Brain magnetic resonance imaging (MRI) only revealed a right frontal ischemic sequelae. Fundus examination was normal without sign of retinitis. Upper endoscopy showed erosive gastritis. Immunostaining of gastric biopsies revealed CMV nuclear inclusions (Fig. 2), suggesting CMV enteritis in the setting of persisting hiccup and acute diarrhea without other documented pathogens.

Renal-dosed intravenous ganciclovir later replaced by oral valganciclovir after improvement of diarrhea was introduced for 3 weeks. CMV viral load became undetectable after 2 weeks. Antibiotic treatment with flucloxacillin (2 g 4/day) was started for 14 days together with local wound treatment for MSSA surgical site infection. Mycophenolate mofetil was transiently interrupted, and prednisone dosage subsequently increased. The patient made a complete clinical and biological recovery. He was discharged with maintenance half-dose valganciclovir therapy for a total duration of 3 months. No recurrence of CMV infection was reported 1 year later.

## Discussion and conclusions

CMV remains a leading pathogen in immunocompromised hosts, either presenting as a viral syndrome with general malaise and cytopenia or as a tissue-invasive disease, affecting lung, central nervous system, gastro-intestinal tract, liver or presenting as retinitis [2]. In immunocompetent adults CMV infection commonly presents itself as a self-limited disease, an asymptomatic infection or a mononucleosis-like syndrome with diffuse myalgia, prolonged fever and fatigue [3]. Recently, cases of severe disease in immunocompetent adults with co-morbid conditions such as cancer or renal insufficiency [4], or even without such comorbidities [5], have been described. Gastrointestinal tract is mostly affected, with colitis being the most frequent manifestation of disease [2]. Specific anti-viral treatment in this situation is controversial [2], although different case series tend to show a more favorable outcome with early treatment [2, 4, 5].

In organ transplant recipients, CMV disease causes important morbidity and mortality, and may compromise transplant success due to indirect effect in triggering transplant rejection [6]. Current guidelines recommend pre-transplant donor and recipient serology, in order to determine post-transplant strategy either with systemic prophylaxis or preemptive therapy, with practices varying depending on centers. Patients with a D+/R− serostatus are at higher risk [1]. Universal prophylaxis is defined as systematic antiviral medication for a defined period of time following transplantation (usually 3–6 months). Preemptive therapy involves serial viral load monitoring and antiviral treatment initiation once replication attains a predefined threshold, before clinical symptoms develop.

Studies comparing both strategies showed conflicting results, maybe due to varying frequencies of viral load measurements [6, 7]. Currently both strategies are accepted for liver and renal transplantation [1]. Hybrid approaches have also been used, such as preemptive strategy after discontinuation of antiviral prophylaxis [8]. In case of patients with D−/R− serostatus, no prophylaxis is recommended as these patients are considered at low risk for CMV disease [1].

Valganciclovir or iv ganciclovir in case of severe disease or anticipated poor oral absorption are currently recommended for treatment of CMV disease. A treatment duration of at least 2 weeks guided by weekly monitoring of CMV viral load until negative, and clinical evolution is standard practice [1]. Higher initial viral load [7], higher intensity of immunosuppression [9], and gastrointestinal disease [1] are factors that may justify longer treatment duration due to a higher risk of relapse.

After successful treatment of CMV disease, secondary prophylaxis may be prescribed based on presumed risk factors for recurrent disease [1]. Duration of secondary prophylaxis ranges from 1 to 3 months and may be prolonged in severe cases with other risk factors, such as a high net state of immunosuppression or concomitant treatment of acute rejection [1].

Our patient developed a primary CMV infection 12 years after renal transplant without recent increase in immunosuppression, but in the setting of severe renal insufficiency, with a predominating gastrointestinal presentation, and a rapidly favorable clinical evolution under antiviral treatment. In such cases of very late occurring primary infection, with a low net state of immunosuppression, but with other risk factors for recurrence, the impact of secondary prophylaxis is unknown.

In a case series of 2489 renal transplant patients followed between 1985 and 2007, late onset CMV infection was defined as occurring more than 1 year after transplantation [10]. Of 2489 patients, 3.1% (77 patients) developed late onset CMV infection, some of them more than 10 years after transplantation. Although D+/R− patients were overrepresented, 15% of late-onset CMV disease occurred in D−/R− recipients. Per protocol, all episodes were treated with a 2-week course of induction therapy, followed by 6 weeks of half-dose maintenance therapy. These very late-onset CMV diseases were also associated with impaired outcomes, such as decreased patient and graft survival, perhaps because of delayed diagnosis of these predominantly primary infections.

In this case series, as many as 34% of all late onset CMV infections had recurrent infections. No further analyses of differences between these patients and those without recurrences were discussed.

Another case report described a primary CMV infection occurring 14 years post heart transplant [11]. This D−/R− 44 year-old-patient under prednisone, cyclosporine and mycophenolate mofetil was hospitalized for gastro-intestinal CMV disease. He was initially treated with intravenous ganciclovir followed by oral valganciclovir twice daily. The viral load remained detectable for 5 weeks although no resistance to treatment was detected. The evolution of this patient was remarkable for an immunological complication (chronic inflammatory demyelinating polyneuropathy). The patient remained on valganciclovir maintenance therapy for more than 3 months, and the prednisone dosage was notably increased.

Primary infections leading to late-onset CMV disease in transplant patients remain rare, and few publications are available to guide clinical management. Recurrent disease has been described in as many as one-third of these patients [10]. Due to the paucity of published data, the impact of secondary prophylaxis in this particular group of patients remains unknown. If required, duration of secondary prophylaxis might vary according to other risk factors associated with recurrence of CMV infection [1]. Larger case series in the future with careful description of clinical presentation, evolution under antiviral treatment, and evaluation of the patient’s net state of immunosuppression at time of infection, might help to identify patients for whom secondary prophylaxis could be safely avoided.

## Abbreviations

CMV: cytomegalovirus
D: donor
R: recipient
MSSA: *methicillin*- *susceptible*-*Staphylococcus aureus*
MRI: magnetic resonance imaging
